# Supplementary material for: Proteome census upon nutrient stress reveals Golgiphagy membrane receptors
Source: Nature. 2023 Sep 27;623(7985):167–74. doi: 10.1038/s41586-023-06657-6 (PMC10620096; doi:10.1038/s41586-023-06657-6)
Supplement: Supplementary file 1 — Uncropped gel images for immunoblots and flow cytometry gating examples for Keima flux measurements. [file 41586_2023_6657_MOESM1_ESM.pdf]

nature portfolio

https://doi.org/10.1038/s41586-023-06657-6

Supplementary information

Proteome census upon nutrient stress reveals Golgiphagy membrane receptors

In the format provided by the authors and unedited

Uncropped Blots (Hickey et al.)

Extended data fig 1a, b, c

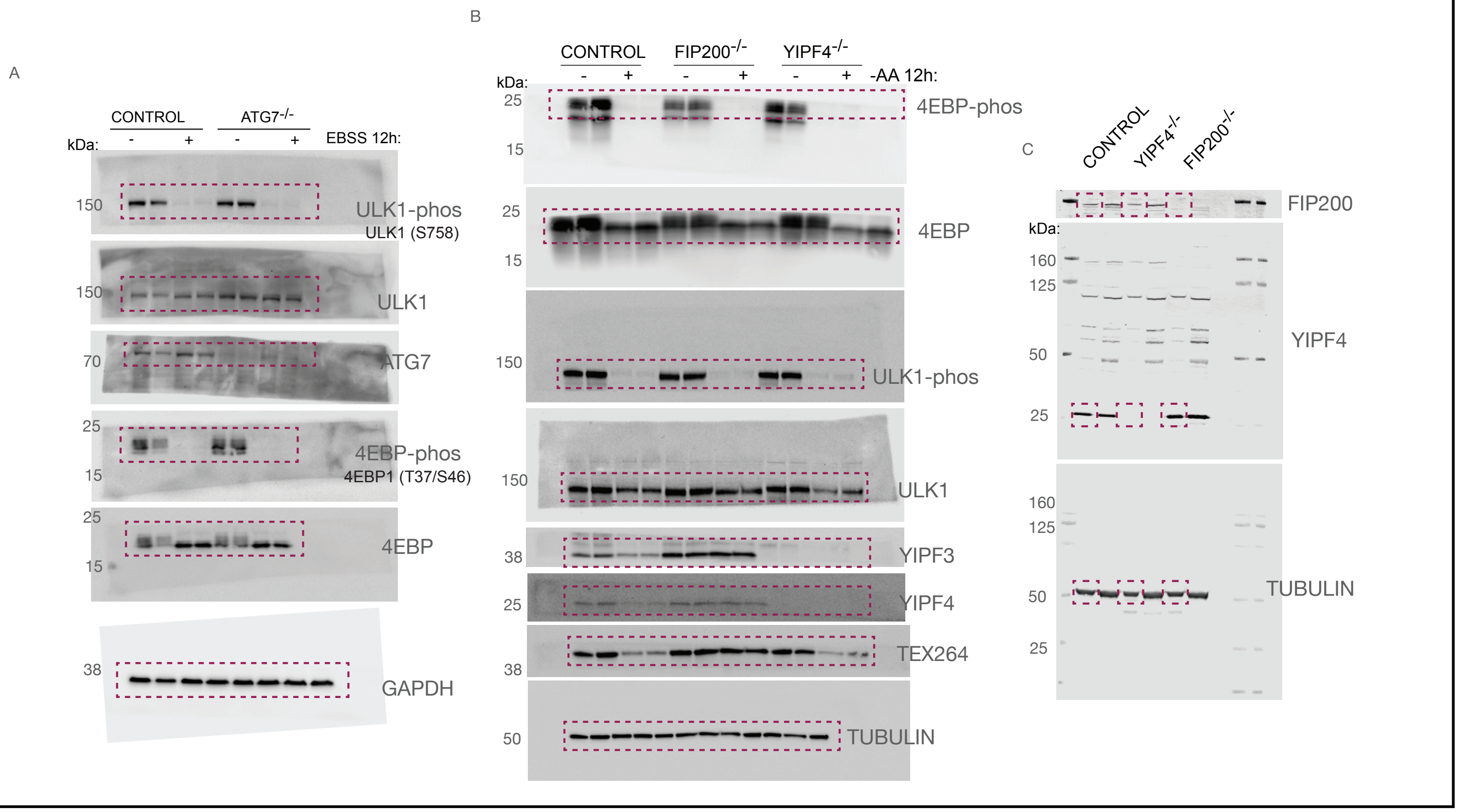

Figure 3f

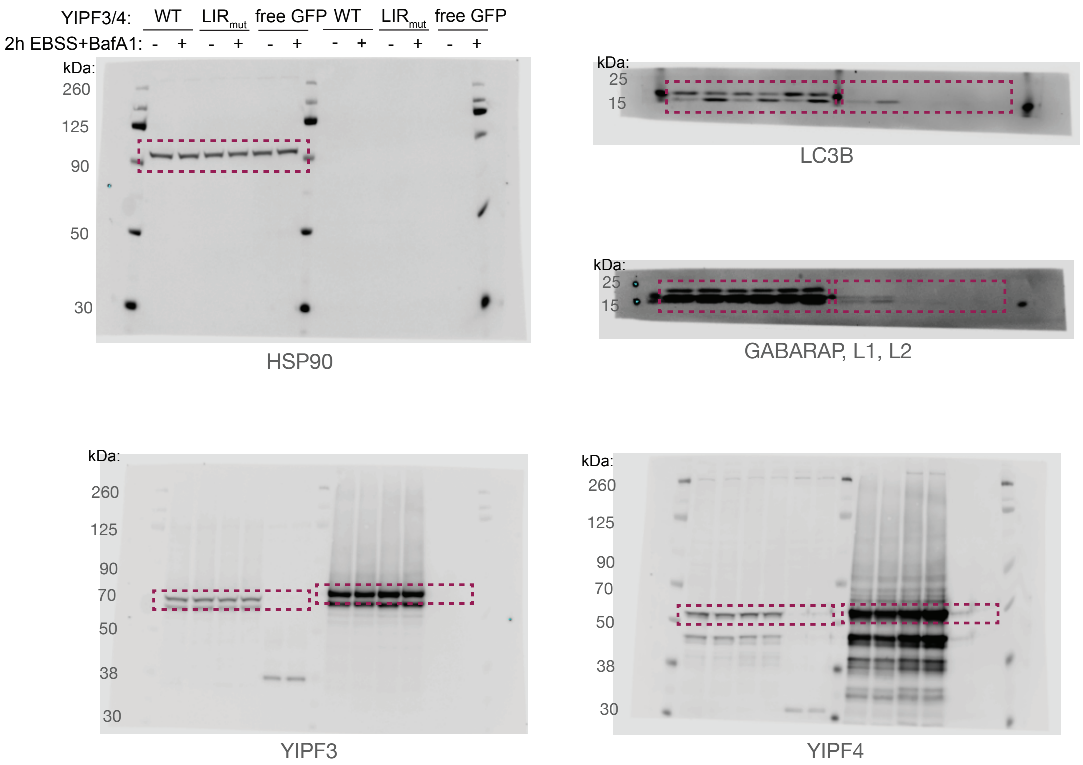

YIPF3/4: WT LIRmut free GFP WT LIRmut free GFP
2h EBSS+BafA1: - + - + - + - + - + - +
kDa:
260
125
90
50
30
HSP90
kDa:
25
15
LC3B
kDa:
25
15
GABARAP, L1, L2
kDa:
260
125
90
70
50
38
30
YIPF3
kDa:
260
125
90
70
50
38
30
YIPF4

Extended data fig 5a

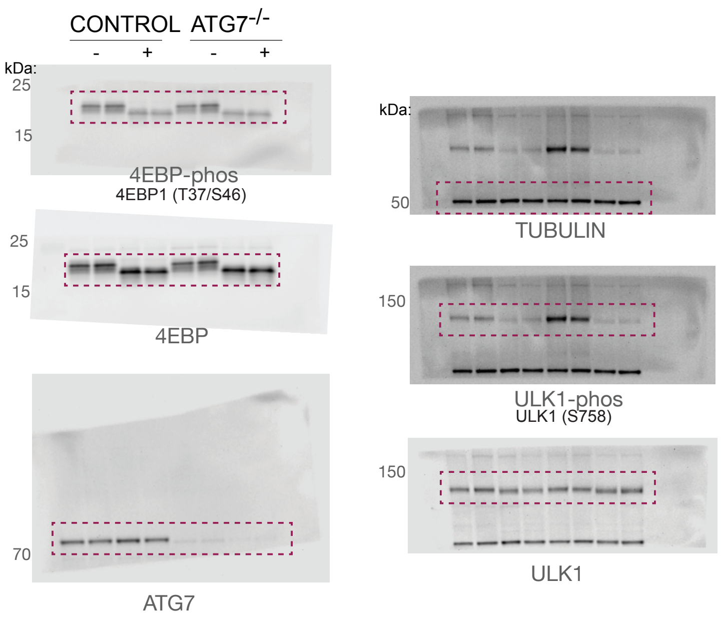

Extended data fig 7a

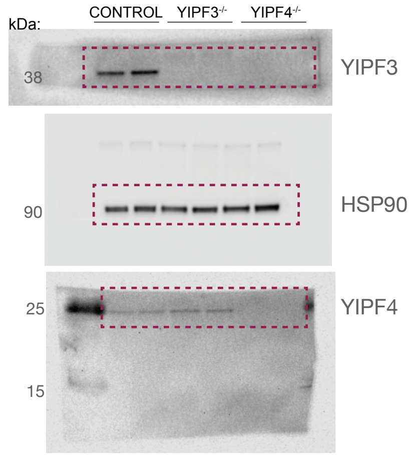

Extended data fig 8b

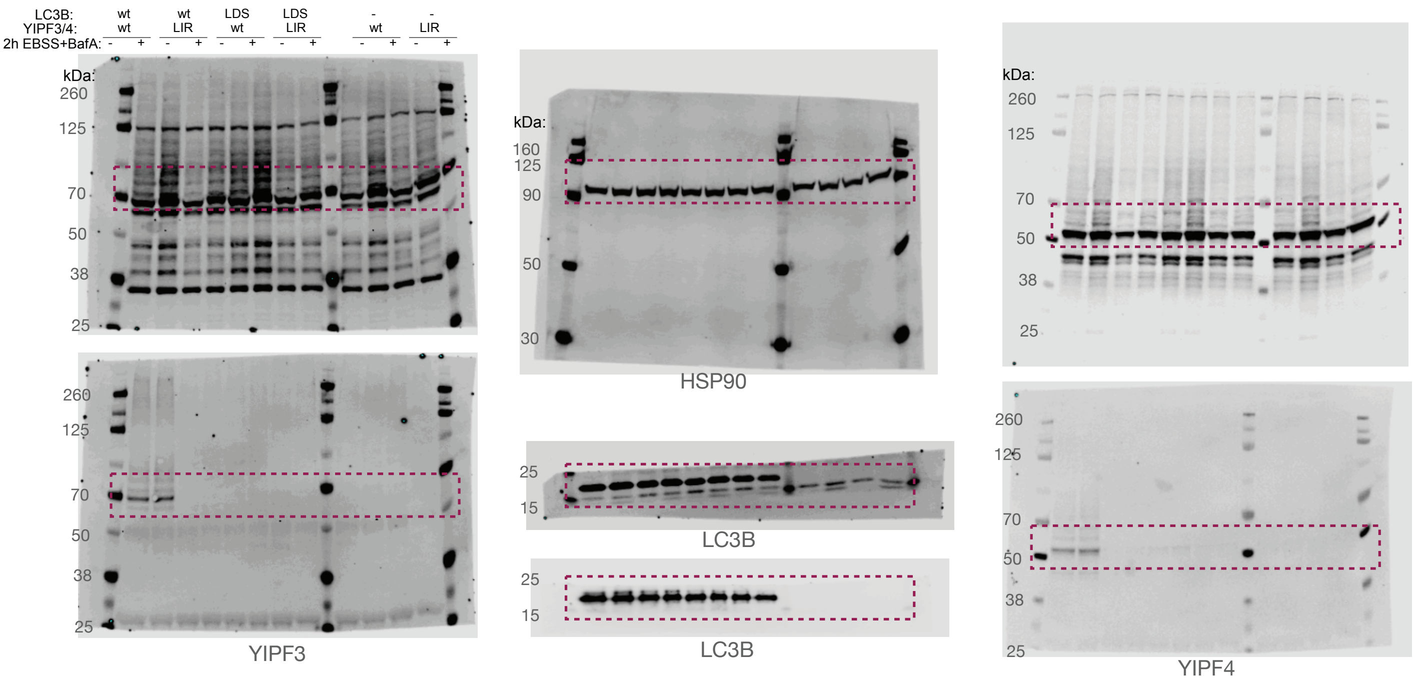

Extended data fig 8c

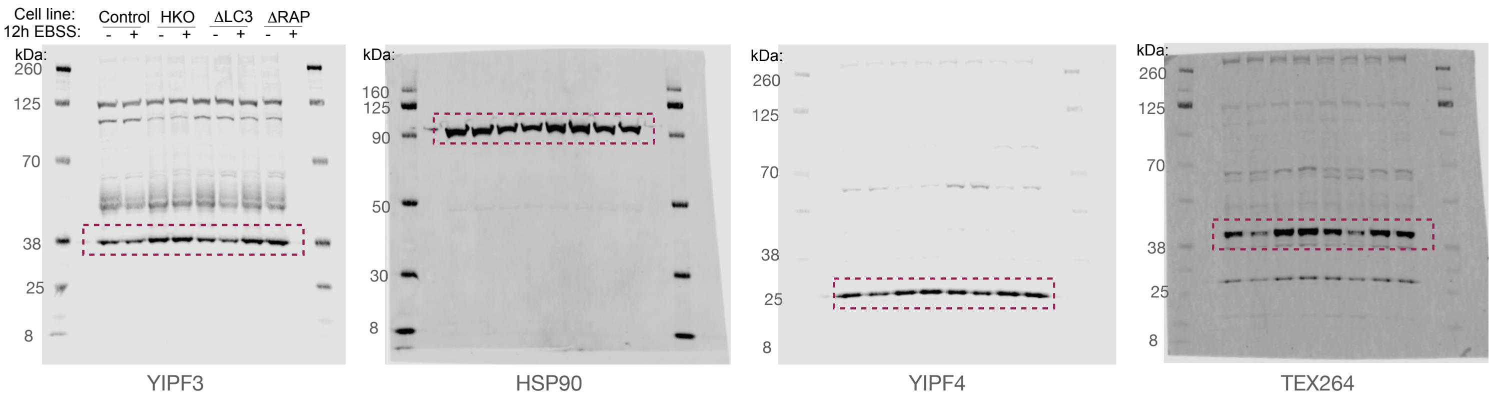

Extended data fig 9a

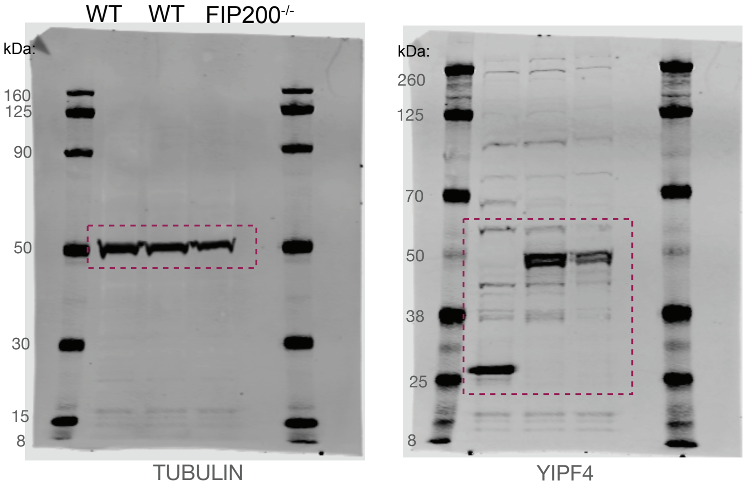

Extended data fig 9f

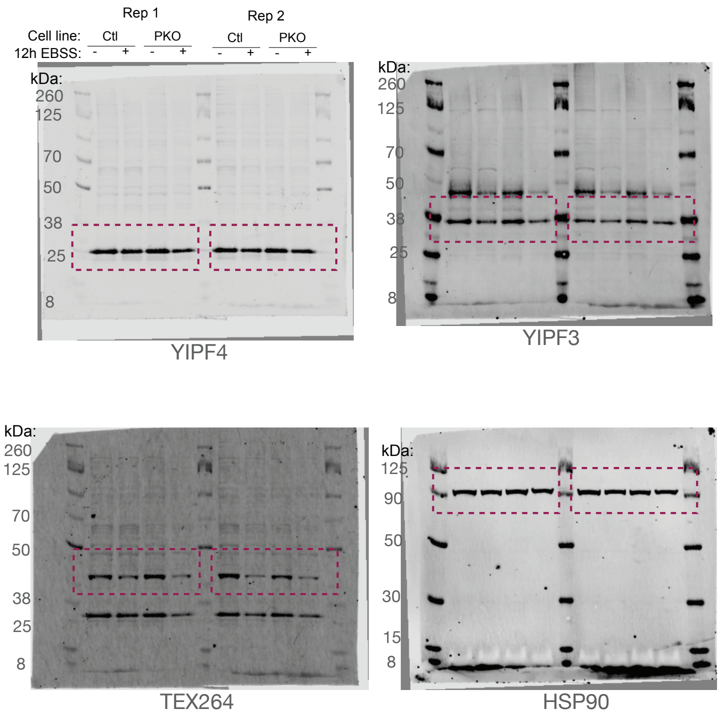

Extended data fig 10c

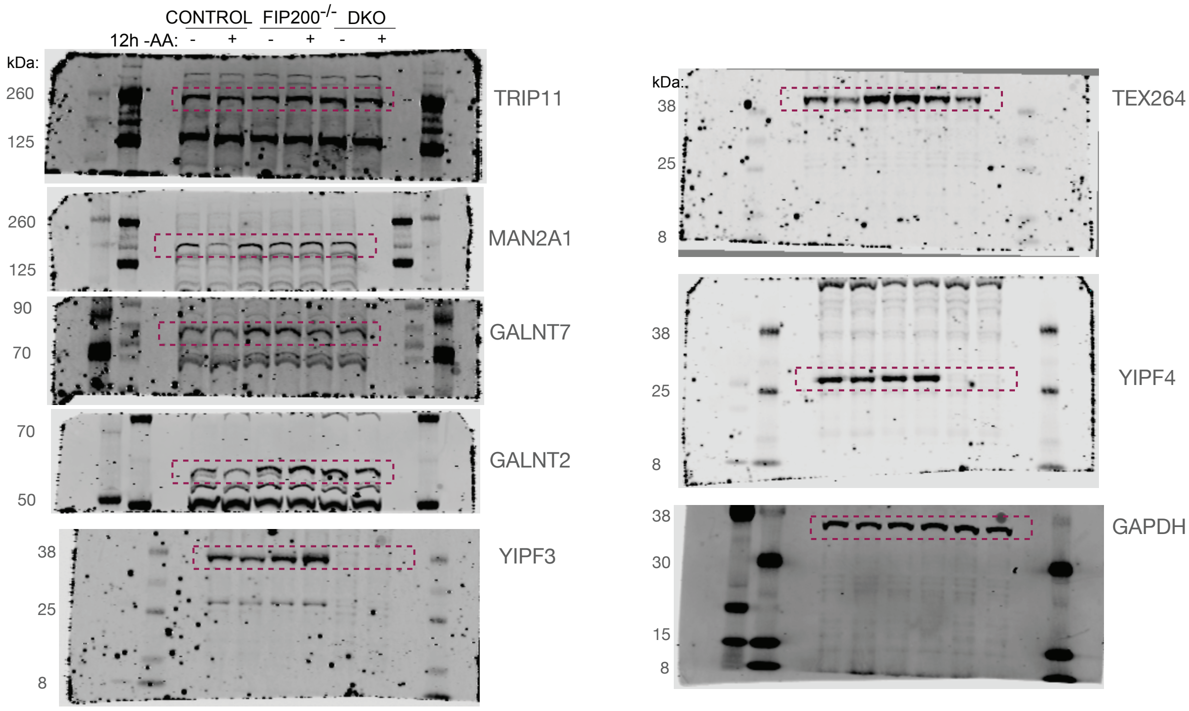

Extended data fig 10d- used for quantification

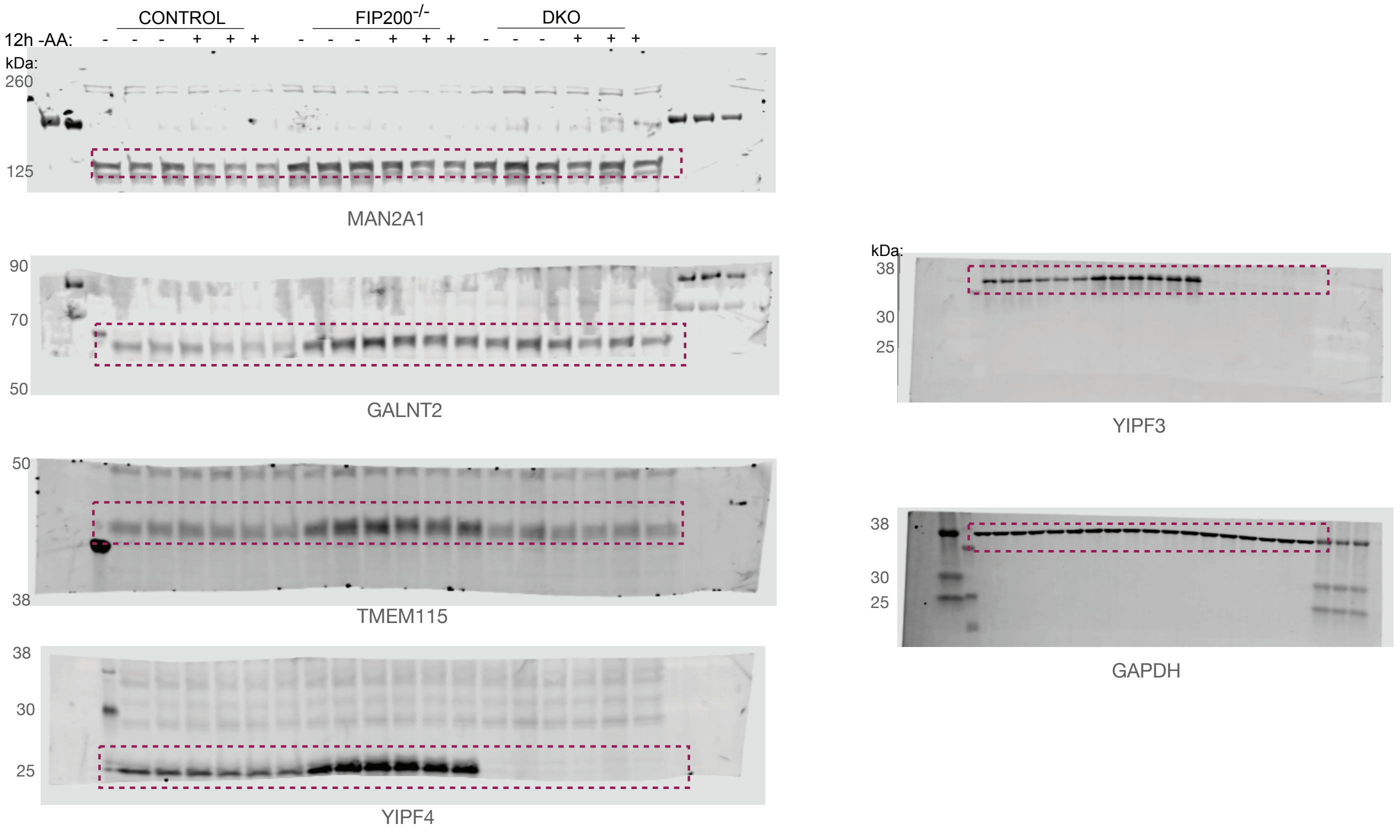

Extended data fig 11b

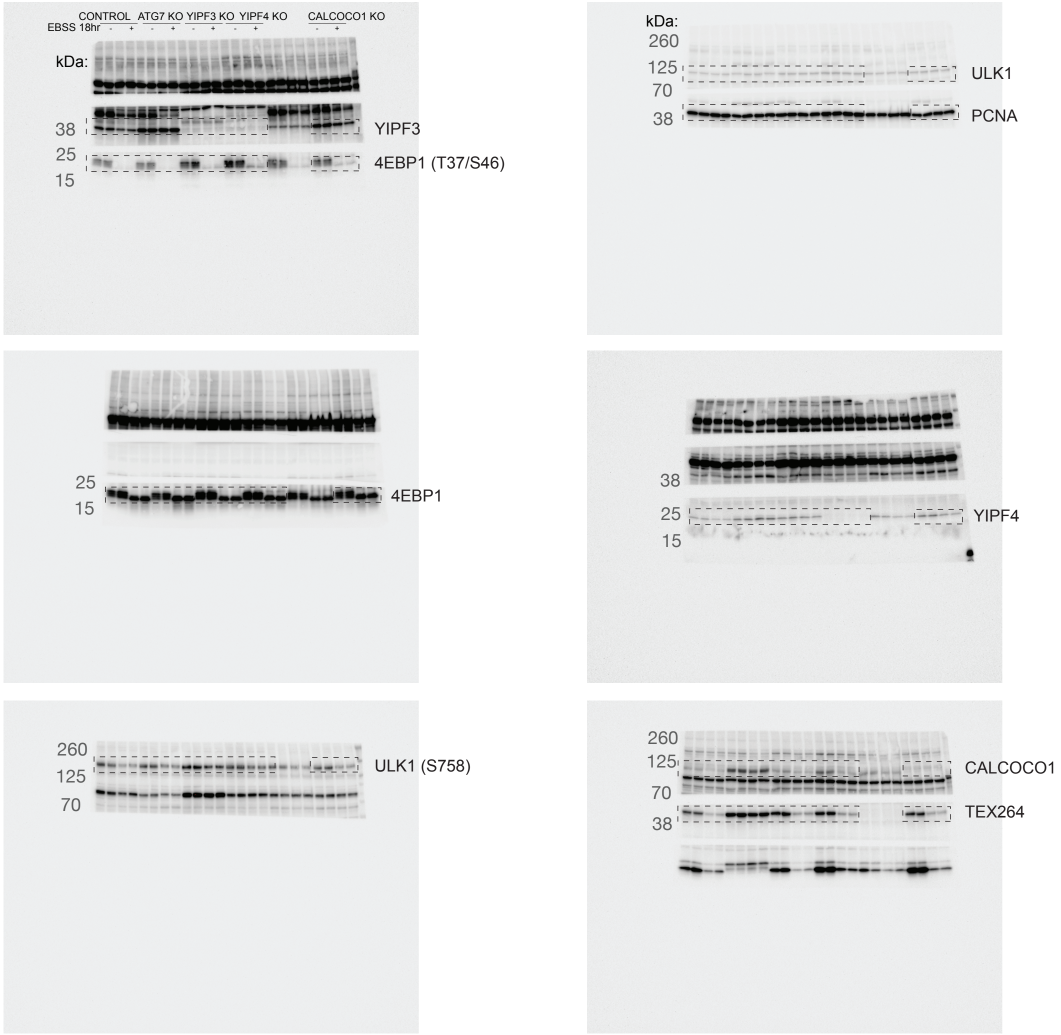

Example of Keima flow cytometry gating strategy:

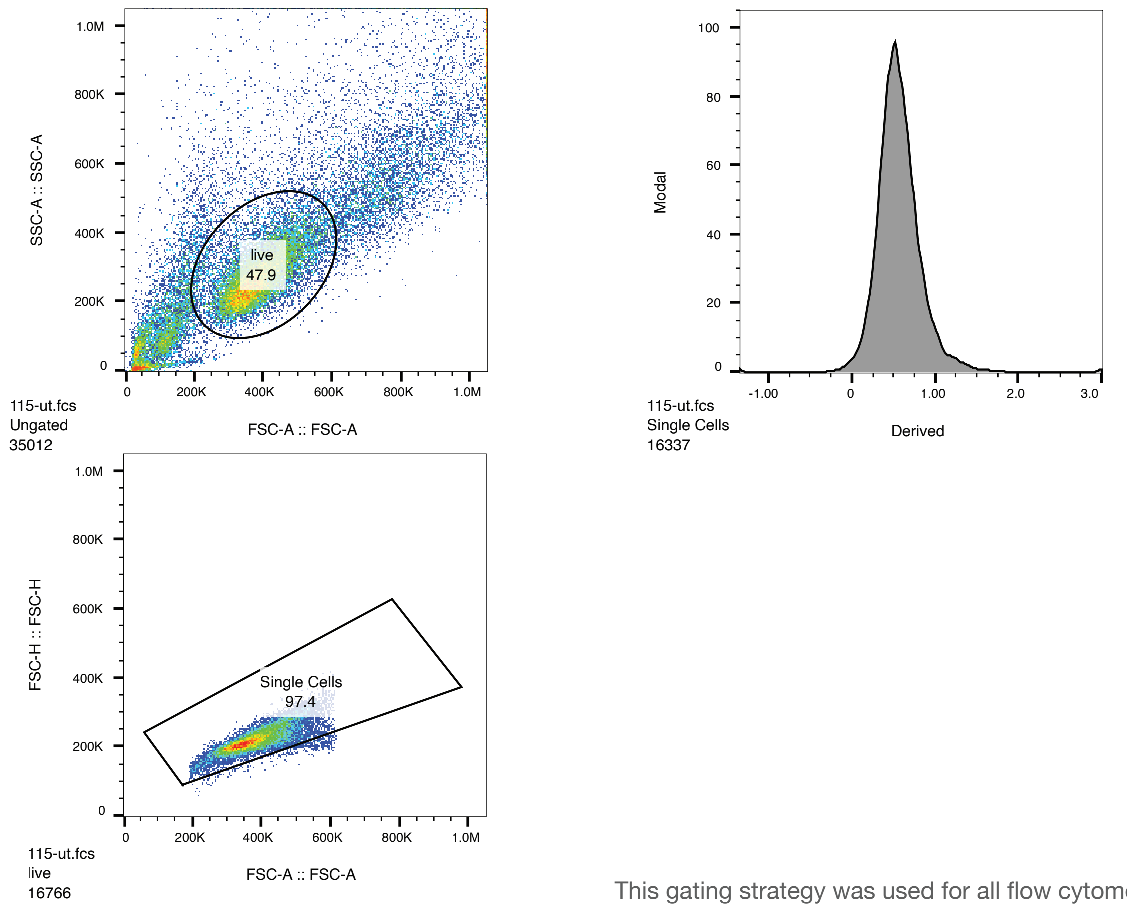

This gating strategy was used for all flow cytometry experiments:
Figure 2a, d; Extended Data Figure 10f
