# Supplementary material for: Proteome census upon nutrient stress reveals Golgiphagy membrane receptors
Source: Nature. 2023 Sep 27;623(7985):167–74. doi: 10.1038/s41586-023-06657-6 (PMC10620096; doi:10.1038/s41586-023-06657-6)
Supplement: Supplementary file 2 — Reporting Summary [file 41586_2023_6657_MOESM2_ESM.pdf]

nature research

Corresponding author(s): J. Wade Harper

Last updated by author(s): 8/30/2023

Reporting Summary

Nature Research wishes to improve the reproducibility of the work that we publish. This form provides structure for consistency and transparency in reporting. For further information on Nature Research policies, see Authors & Referees and the Editorial Policy Checklist.

Statistics

For all statistical analyses, confirm that the following items are present in the figure legend, table legend, main text, or Methods section.

n/a	Confirmed	
☐	☒	The exact sample size (n) for each experimental group/condition, given as a discrete number and unit of measurement
☐	☒	A statement on whether measurements were taken from distinct samples or whether the same sample was measured repeatedly
☐	☒	The statistical test(s) used AND whether they are one- or two-sided<br>*Only common tests should be described solely by name; describe more complex techniques in the Methods section.*
☒	☐	A description of all covariates tested
☐	☒	A description of any assumptions or corrections, such as tests of normality and adjustment for multiple comparisons
☐	☒	A full description of the statistical parameters including central tendency (e.g. means) or other basic estimates (e.g. regression coefficient) AND variation (e.g. standard deviation) or associated estimates of uncertainty (e.g. confidence intervals)
☐	☒	For null hypothesis testing, the test statistic (e.g. F, t, r) with confidence intervals, effect sizes, degrees of freedom and P value noted<br>*Give P values as exact values whenever suitable.*
☒	☐	For Bayesian analysis, information on the choice of priors and Markov chain Monte Carlo settings
☒	☐	For hierarchical and complex designs, identification of the appropriate level for tests and full reporting of outcomes
☒	☐	Estimates of effect sizes (e.g. Cohen's d, Pearson's r), indicating how they were calculated

Our web collection on statistics for biologists contains articles on many of the points above.

Software and code

Policy information about availability of computer code

Data collection

Orbitrap Eclipse Tribrid Mass Spectrometer (Cat#FSN04-10000) with FAIMS Pro Interface (#FMS02-10001) - Thermo Fisher Scientific
Orbitrap Fusion Lumos Tribrid MS (Cat#IQLAAEGAAPFADBMBHQ) with or without FAIMS Pro Interface (#FMS02-10001) - Thermo Fisher Scientific
Odyssey CLx Imager LI-COR bioscience
Nikon Ti motorized microscope equipped with a Nikon Plan Apo 100x/1.40 N.A objective lens, and Hamamatsu ORCA-Fusion BT CMOS camera- Nikon
Attune NxT Flow Cytometer (Cat#A28993)- Thermo Fisher Scientific

Data analysis

1. Prism; GraphPad, v9 https://www.graphpad.com/scientific-software/prism/
2. SEQUEST-HT ; Eng et al., (1994) J Am Soc Mass Spectrom. 5 (11): 976-989. Implementation in Proteome Discoverer (v2.3.0.420 – Thermo Fisher Scientific)
3. Comet (v2018.01 rev. 2); Eng, J.K. et al. (2013), Proteomics 13, 22-24.
4. FlowJoTM; Vl0.5.2 https://www.flowjo.com
5. lmageStudiolite V 5.2.5 https://www.licor.com/bio/products/software/image_studio_lite
6. FiJi lmageJ V.2.0.0 https://imagej.net/Fiji
7. Rstudio (1.2.1335) + R(v_4.1.3)
8. Adobe lllustrator(CS5(15.0.0))
9. Monocole, Rad et al., J. Proteome Res. 20, 591-598 (2021)
10. Code and data analysis to generate paper figures can be found on GitHub at https://github.com/harperlaboratory/Golgiphagy.git. All data and data figures can be explored using CARGO (Cellular Autophagy Regulation and GOlgiphagy). CARGO is a ShinyApp interface generated in R and RStudio that can be accessed at https://harperlab.connect.hms.harvard.edu/CARGO_Cellular_Autophagy_Regulation_GOlgiphagy/.

For manuscripts utilizing custom algorithms or software that are central to the research but not yet described in published literature, software must be made available to editors/reviewers. We strongly encourage code deposition in a community repository (e.g. GitHub). See the Nature Research guidelines for submitting code & software for further information.

Data

Policy information about availability of data

All manuscripts must include a data availability statement. This statement should provide the following information, where applicable:

- Accession codes, unique identifiers, or web links for publicly available datasets
- A list of figures that have associated raw data
- A description of any restrictions on data availability

Data Availability
All mass spectrometry data for HeLa and HEK293 cells (155 files) have been deposited to the ProteomeXchange Consortium via the PRIDE repository (http://www.proteomexchange.org/): (Project Accession: PXD038358). Proteomic data for ES cells and iNeurons (15 files) is available on Project Accession: PXD043923. All analyzed proteomic data are in Supplementary Tables 1, 2, 4, 5, 6, 7, 8, and 9. Source codes for figures are provided in Source data Table 1 and all uncropped blots are provided in Supplementary Figure 1. We employed canonical protein entries from the Human reference proteome database in our study (UniProt Swiss-Prot – 2019-01; https://ftp.uniprot.org/pub/databases/uniprot/previous_major_releases/release-2019_01/). LIR motifs were based on the iLIR Autophagy Database (http://repeat.biol.ucy.ac.cy/iLIR/).

Field-specific reporting

Please select the one below that is the best fit for your research. If you are not sure, read the appropriate sections before making your selection.

☒ Life sciences ☐ Behavioural & social sciences ☐ Ecological, evolutionary & environmental sciences

For a reference copy of the document with all sections, see nature.com/documents/nr-reporting-summary-flat.pdf

Life sciences study design

All studies must disclose on these points even when the disclosure is negative.

Sample size	No sample size calculation was done. For proteomics, we chose n=2, 3 or 4 biological replicates given the limitation of the available TMT channels and extensive work in the field has shown that this approach provides the necessary statistical significance. The number of replicates for all TMT experiments is shown in the schematic in the relevant figure. For flow cytometry, we analyzed >10,000 cells with biological triplicate experiments, which showed consistent results throughout the replication. The number of replicates for immunoblotting experiments is provided in the figure legends and is performed in triplicate unless otherwise noted. Confocal imaging experiments were performed in biological triplicate at a minimum (a subset involved five or 6 replicates). The number of data points in each plot represents the number of replicates used. Sample size was determined based on similar studies in this field. e.g. An et al Systematic quantitative analysis of ribosome inventory during nutrient stress. Nature. 2020 Jul;583(7815):303-309. doi: 10.1038/s41586-020-2446-y
Data exclusions	No data were excluded from the analyses.
Replication	We confirm that all attempts at replication were successful. The number of biological replicates is provided for each experiment in the figure legend.
Randomization	No randomization was necessary. Mass spectrometry and biochemistry samples were measured sequentially. Images were automatically acquired for the data analysis by high throughput imaging based methods.
Blinding	No blinding was applied in this study. Blinding was not possible as all samples were analyzed pairwise or multiple samples compared. In all assays in this study the treatment (or different conditions tested) cannot be disguised from the scientist.

Reporting for specific materials, systems and methods

We require information from authors about some types of materials, experimental systems and methods used in many studies. Here, indicate whether each material, system or method listed is relevant to your study. If you are not sure if a list item applies to your research, read the appropriate section before selecting a response.

Materials & experimental systems

n/a	Involved in the study
☐	☒ Antibodies
☐	☒ Eukaryotic cell lines
☒	☐ Palaeontology
☒	☐ Animals and other organisms
☒	☐ Human research participants
☒	☐ Clinical data

Methods

n/a	Involved in the study
☒	☐ ChIP-seq
☐	☒ Flow cytometry
☒	☐ MRI-based neuroimaging

Antibodies

Antibodies used

ATG7 (Cell Signaling Technology, 8558S; RRID:AB_10831194; dilution 1:1000). Lot: 4
FIP200 (Proteintech, 17250-1-AP; RRID: AB_10666428; dilution 1:1000). Lot: 00048639
LC3B (D11) XP(R) (Cell Signaling Technology, 3868; AB_2137707; dilution 1:1000). Lot: 6
ULK1 (Cell Signaling Technology 8054; RRID:AB_11178668; dilution 1:1000). Lot:6
Phospho-ULK1 (ser757) (Cell Signaling Technology 14202; RRID:AB_2665508; dilution 1:1000). Lot: 5
4E-BP1 (Cell Signaling Technology 9644; RRID:AB_2097841; dilution 1:1000). Lot: 12
Phospho-4E-BP1 (Thr37/46) (Cell Signaling Technology 2855; RRID:AB_560835; dilution 1:1000). Lot: 26
TEX264 (Sigma, HPA017739; RRID:AB_1857910; dilution 1:1000). Lot: 000012723
Tubulin (Abcam, ab131205; RRID: AB_11156121; dilution 1:1000). Lot: GR3251127-3
YIPF3 (Invitrogen PA566621; RRID:AB_2664704; dilution 1:1000). Lot: YF3956672B
YIPF4 (Sino Biological 202844-T46; dilution 1:1000) Lot: HD12JL0934
HSP90 (Santa Cruz Biotechnology sc-69703; AB_2121191; dilution 1:1000). Lot: J2721
CALCOCO1 (Abclonal A7987; RRID:AB_2768684; dilution 1:1000). Lot: 0036240101
LAMP1 (Cell Signaling Technology 9091; RRID:AB_2687579; dilution 1:1000). Lot: 5
GOLGB1/Giantin (abcam ab37266; RRID:AB_880195; dilution 1:1000) Lot: GR3452700-3
GOLGA2 (Proteintech 11308; RRID:AB_2919024; dilution 1:1000). Lot: 00039607
PCNA (Santa Cruz PC10; sc-56 RRID:AB_628110; dilution 1:1000). Lot: L3015
IRDye 800CW Goat anti-Rabbit IgG H+L (LI-COR, 926-32213; AB_621848; dilution 1:10000). Lot: D21104-25
IRDye 680 RD Goat anti-Mouse IgG H+L (LI-COR, 926-680; RRID:AB_10956588; dilution 1:10000). Lot: D00825-11
Goat anti-Rabbit IgG, HRP-linked IgG (Cell Signaling Technology 7074P2, RRID: AB_2099233 dilution 1:10000). Lot: 28
Goat anti-Rabbit IgG HRP conjugate (Bio-Rad 1706515; RRID:AB_11125142; dilution 1:10000). Lot: 64559210
Goat anti-Mouse IgG HRP conjugate Bio-Rad 1706516; RRID:AB_11125547; dilution 1:10000). Lot: 64526160

Validation

1. FIP200, YIPF4, YIPF3, CALCOCOl, ATG7 antibody specificity determined by CRISPR deletion or tagging of endogenous gene (see figures Extended Data Fig. 1a-c, 7a).
2. Specificity of Tex264 was determined previously using TEX264-/- cells (Mol Cell, 74, 891 (2019)).
3. PCNA was validated using knockout cells (Biotechniques. 2017;62:80-82).
4. LAMP1 (D2D11) XP® Rabbit mAb recognizes endogenous levels of total LAMP1 protein, as reported by the vendor (https://www.cellsignal.com/products/primary-antibodies/lamp1-d2d11-xp-rabbit-mab/9091).
5. HSP90 antibody has is applicable for WB, RIP, IP, IHC, IF, FC, CoIP, ELISA and shows reactivity with human samples. (https://www.ptglab.com/products/HSP90-Antibody-60318-1-Ig.htm).
6. Tubulin antibody has been shown to be excellent as a loading control antibody and reacts with human tubulin (https://www.abcam.com/products/primary-antibodies/alpha-tubulin-antibody-dm1a-loading-control-ab7291.html).
7. Giantin antibody is optimized for immunofluorescence and reacts with human giantin (https://www.abcam.com/products/primary-antibodies/giantin-antibody-9b6-golgi-marker-ab37266.html).
8. GOLGB1 Positive WB detected in HeLa and HEK293 cells (https://www.ptglab.com/products/GOLGA2,GM130-Antibody-11308-1-AP.htm).
9. 4E-BP1 (53H11) Rabbit mAb has been characterized by the vendor and detects endogenous levels of total human 4E-BP1 protein (https://www.cellsignal.com/products/primary-antibodies/4e-bp1-53h11-rabbit-mab/9644).
10. Phospho-4E-BP1 (Thr37/46) (236B4) Rabbit mAb has been characterized by the vendor and detects endogenous levels of 4E-BP1 only when phosphorylated at Thr37 and/or Thr46.(https://www.cellsignal.com/products/primary-antibodies/phospho-4e-bp1-thr37-46-236b4-rabbit-mab/2855).
11. GOLGA2 has been shown by the vendor to work for WB and IF applications and shows reactivity with human samples (https://www.ptglab.com/products/GOLGA2,GM130-Antibody-11308-1-AP.htm).
12. ULK1 (D8H5) Rabbit mAb recognizes endogenous levels of total human ULK1 protein, as validated by the vendor. (https://www.cellsignal.com/products/primary-antibodies/ulk1-d8h5-rabbit-mab/8054).
13. Phospho-ULK1 (D7O6U) Rabbit mAb recognizes endogenous levels of ULK1 protein only when phosphorylated at Ser758 of human ULK1 and ser757 in mouse ULK1 (https://www.cellsignal.com/products/primary-antibodies/phospho-ulk1-ser757-d7o6u-

rabbit-mab/14202).

Eukaryotic cell lines

Policy information about cell lines

Cell line source(s)	Human: HEK293 ATCC CRL-1573; RRID:CVCL_0045<br>Human: HeLa ATCC CCL-2; RRID: CVCL_0030<br>Human ES cells (clone H9), WiCell
Authentication	ATCC and WiCell preforms quality testing to ensure authentication of cell lines using Short Tandem Repeat (STR) analysis. Additionally, WiCell performs karyotyping on ES cells. No additional authentications were preformed. karyotyping (GTG-banded karyotype) of HEK293 and HeLa cells (from ATCC) was also performed by the cytogenomics core laboratory at Brigham and Women's Hospital.
Mycoplasma contamination	All cell lines were found to be free of mycoplasma using Mycoplasma Plus PCR assay kit (Agilent).
Commonly misidentified lines<br>(See ICLAC register)	none

Flow Cytometry

Plots

Confirm that:

☒ The axis labels state the marker and fluorochrome used (e.g. CD4-FITC).

☒ The axis scales are clearly visible. Include numbers along axes only for bottom left plot of group (a 'group' is an analysis of identical markers).

☒ All plots are contour plots with outliers or pseudocolor plots.

☒ A numerical value for number of cells or percentage (with statistics) is provided.

Methodology

Sample preparation	No tissue processing were used.
Instrument	Attune NxT Flow Cytometer- Thermo Fisher Scientific
Software	FlowJoTM; V10.5.2 https://www.flowjo.com
Cell population abundance	10,000 cells were recorded per replicate
Gating strategy	1. live cells were gated by SSC1 hight/FSC1 hight (G1) followed by live cells by SSC1 hight/SSC1-width (G2). 2. Keima signal was measured by 405ex/620(20)em and 561ex/620(20)em and data exported to prism for ratio-metric calculation.

☒ Tick this box to confirm that a figure exemplifying the gating strategy is provided in the Supplementary Information.
